# Unmanned aerial vehicles for surveillance and control of vectors of malaria and other vector-borne diseases

**DOI:** 10.1186/s12936-022-04414-0

**Published:** 2023-01-20

**Authors:** Frank Mechan, Zikmund Bartonicek, David Malone, Rosemary Susan Lees

**Affiliations:** 1 Department of Vector Biology, Liverpool School of Tropical Medicine, Liverpool, L3 5QA UK; 2 Innovative Vector Control Consortium (IVCC), Liverpool School of Tropical Medicine, Liverpool, L3 5QA UK; 3 Bill and Melinda Gates Foundation (BMGF), 500 5th Ave N, Seattle, WA 98109 USA; 4 Innovation to Impact (I2I), Liverpool School of Tropical Medicine, Liverpool, L3 5QA UK

**Keywords:** Unmanned aerial vehicle (UAV), Unmanned aerial system (UAS), Mosquito control, Surveillance, Vector-borne diseases, Drones, Public health

## Abstract

The use of Unmanned Aerial Vehicles (UAVs) has expanded rapidly in ecological conservation and agriculture, with a growing literature describing their potential applications in global health efforts including vector control. Vector-borne diseases carry severe public health and economic impacts to over half of the global population yet conventional approaches to the surveillance and treatment of vector habitats is typically laborious and slow. The high mobility of UAVs allows them to reach remote areas that might otherwise be inaccessible to ground-based teams. Given the rapidly expanding examples of these tools in vector control programmes, there is a need to establish the current knowledge base of applications for UAVs in this context and assess the strengths and challenges compared to conventional methodologies. This review aims to summarize the currently available knowledge on the capabilities of UAVs in both malaria control and in vector control more broadly in cases where the technology could be readily adapted to malaria vectors. This review will cover the current use of UAVs in vector habitat surveillance and deployment of control payloads, in comparison with their existing conventional approaches. Finally, this review will highlight the logistical and regulatory challenges in scaling up the use of UAVs in malaria control programmes and highlight potential future developments.

**Graphical Abstract:**

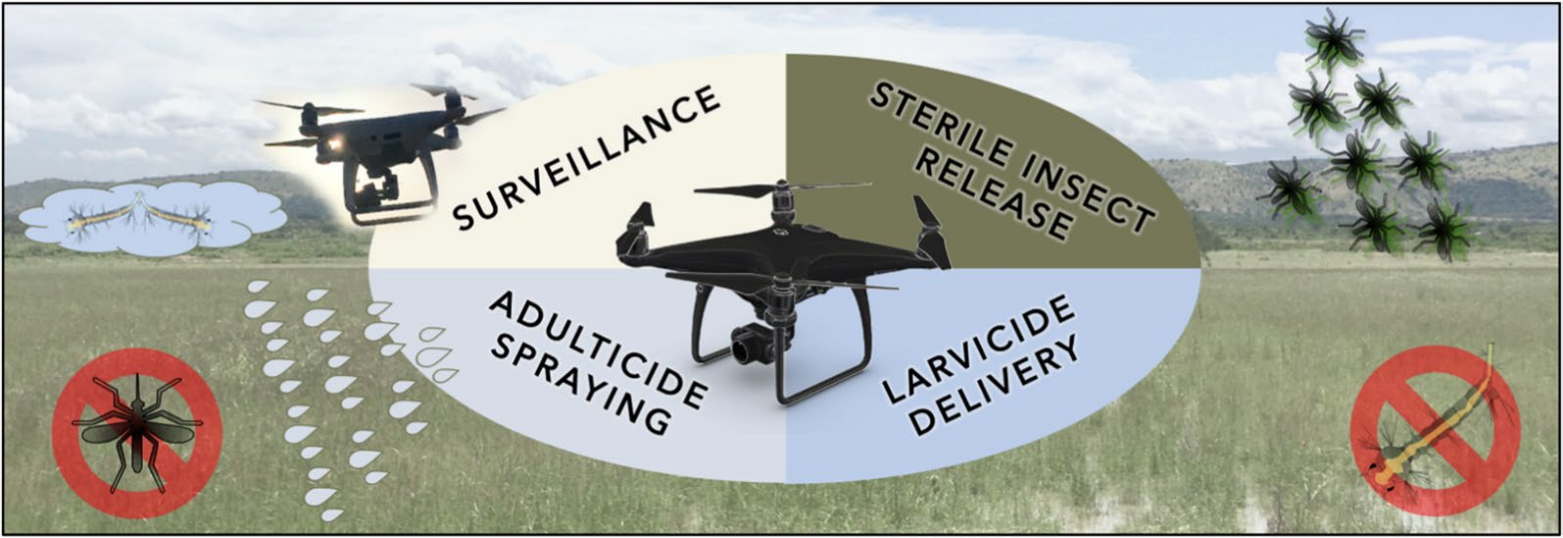

## Background

**Fig. 1:**
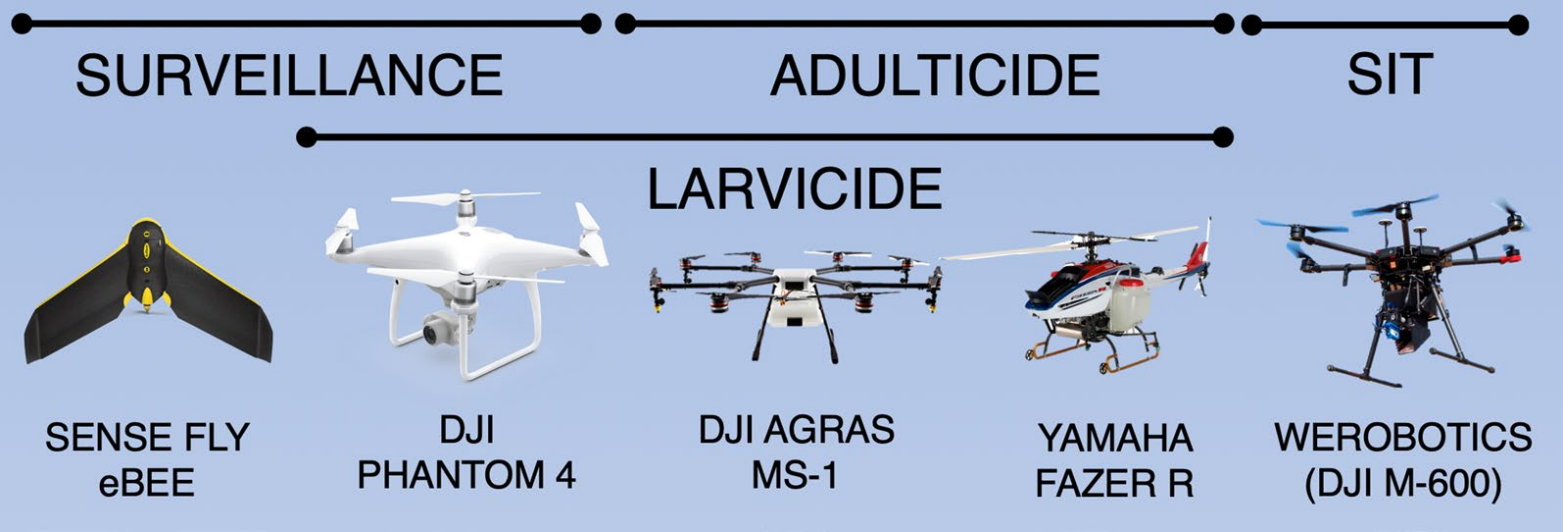
Examples of UAVs used in vector control and their applications (indicated by bars on top). SIT (Sterile Insect Technique) refers to release of sterile insects

Vector-borne diseases (VBDs) are a leading cause of global morbidity and mortality, claiming over 700,000 lives annually with over half of the world’s population at risk of infection by at least one of these diseases [1]. In the case of malaria, primary vector control strategies are the distribution of insecticide-treated nets (ITNs) and indoor residual spraying (IRS) but progress in reducing clinical cases is stagnating, with concern that the World Health Organization (WHO) goal of reducing malaria mortality rates by at least 90% by 2030 (compared with 2015 rates) will not be met [2]. This slow progress against malaria is associated with the challenges of insecticide resistance in vectors and changes in land use that may bring human populations into greater contact with vectors [3]. Consequently, it is essential for malaria researchers and control programmes to focus on novel technologies that aid the surveillance of vectors and the delivery of control agents, with Unmanned Aerial Vehicles (UAVs) being one of the promising possible additions to the toolkit [4]. The use of UAVs has seen a considerable expansion from limited military use to their being utilized in a range of scientific and industrial applications, including agricultural remote sensing [5–7], response to and prevention of pest outbreaks [8, 9], zoonosis control [10], humanitarian emergency response [11, 12], public health [13] and species monitoring for conservation [14, 15]. The idea of using UAVs in malaria control has been postulated for many years [4, 16]. The key capabilities of UAVs are mobility and vantage point; aerial vehicles can rapidly transport sensors or a deployable payload (the cargo or equipment being carried by the UAV) over difficult terrain as well as obtain a bird’s eye view of an area of interest [17] (Fig. 1). While existing technologies and methodologies may already have the means to survey habitats and deploy insecticides in principle, the emergence of relatively inexpensive commercial UAVs allow vector control programmes to achieve these objectives at an unprecedented speed and scale.

The terminology for remotely operated unmanned aircraft is not entirely unified, potentially leading to confusion. While the general public knows UAVs best by the term “drones”, aviation agencies tend to use different names. The International Civil Aviation Organisation uses the acronym RPAS, standing for Remotely Piloted Aircraft System, to describe an unmanned aircraft that is not autonomous, Unmanned Aircraft (UA) for the aircraft itself without any remote control systems, and the term Unmanned Aerial Systems (UAS) for systems that do not necessarily require an operator [18]. As most scientific literature uses the term UAV, this term will also be used in this review. There are two main broad categories of applications for which UAVs can be utilized in combating VBDs. Firstly, the systems can serve as a surveillance tool (gathering entomologically or epidemiologically relevant data) [19, 20] using a variety of onboard sensors such as visible spectrum (wavelengths that the human eye can perceive) or infrared (wavelengths longer than the human eye can perceive, which can be used to remotely determine the temperature of objects) cameras [21, 22]. Secondly, UAVs can be used as a means of delivering vector control interventions to a target site (larvicides, insecticides, or mosquitoes modified to disrupt the vector population) [23, 24]. This structured review of the literature summarizes the existing application of UAV technology for habitat surveillance and the delivery of interventions in the context of malaria control, as well as the use of UAVs in the context of other VBDs where techniques could be readily applied to malaria programmes.

As methodologies existed for these applications prior to the introduction of UAVs, this review assesses the strength and limitations of UAVs compared to conventional approaches. Additionally, the review will summarize the broad design considerations in choosing the appropriate UAV, outlining the advantages of different design categories for vector control applications. Finally, this review will discuss the current regulations on the use of UAVS for vector control, highlighting the challenges and opportunities for scaling up their use.

## Searching the literature

In September 2022, a literature search was conducted using the PubMed database using the search term: “(drones OR aerial vehicle) AND (disease OR vector)”. This search returned 396 articles, which were then manually screened for relevance. Publications were selected if they assessed the use of UAVs to survey vector habitats, deploy insecticides, or release biological control agents. A total of 33 publications were selected for inclusion. Primary sources where UAVs were used for vector control activities are summarized in Table 1.

**Table 1:** Summary of key literature cited

Authors	Setting	Drone type	VBDs targeted	Purpose/outcome
Aragão et al. (2020)	*In silico*/Paraná, Brazil	Not described	Dengue	Drone selection for habitat mapping
Carrasco-Escobar et al. (2019)	Maynas, Peru	DJI Phantom 4 Pro	*P. vivax*/*P. falciparum*	Habitat mapping
Chamberlin et al. (2020)	Senegal River Basin, Senegal	DJI Phantom 4	Schistosomiasis	
Hardy et al. (2017)	Zanzibar	DJI Phantom 3	*P. falciparum* malaria	
Stanton et al. (2021)	Malawi	DJI Phantom 4 Pro	*P. falciparum* malaria	
Valdez-Delgado et al. (2021)	Chiapas, Mexico	DJI Phantom 4 Pro	Dengue/Zika/Chikungunya	
Sarira et al. (2020)	South Australia	Not described	All mosquito-borne diseases	Image processing for habitat mapping
Johnson et al. (2020)	Pannikin Island, Australia	DJI Phantom 4/DJI Mavic Pro	Ross River Virus/Barmah Forest Virus	Habitat mapping/*Bacillus thuringiensis* deployment
Wood et al. (2019)	Senegal River Basin, Senegal	DJI Phantom 4	Schistosomiasis	Habitat mapping/risk mapping
Fornace et al. (2014)	Sabah, Malaysia	Sensefly eBee (fixed wing)	*P. knowlesi* malaria	Risk mapping
Mukabana et al. (2022)	Zanzibar, Tanzania	Agras MG-1 S	*P. falciparum* malaria	Larvicide spraying
Li et al. (2016)	China	Not described	Dengue	Adulticide misting
Bouyer et al. (2020)	Juazeiro, Brazil	DJI M600 Pro	Dengue/Yellow fever/ Zika	Sterile male release
Marina et al. (2022)	Chiapas, Mexico	DJI M600	Dengue/Yellow fever/ Zika	

## UAV use in vector habitat surveillance

UAVs are commonly used for mapping in conservation, agriculture, invasive species detection and other areas [14, 15, 25], with their use in the detecting and controlling mosquito-borne disease increasingly investigated in recent years [19, 22, 26–28]. The abundance and distribution of malaria transmitting mosquitoes is dependent on the availability of water bodies to act as breeding sites [29]. Larval Source Management (LSM) is a malaria intervention that aims to limit human exposure to mosquito bites by reducing the availability of these breeding sites [30, 31]. The visual identification of breeding sites and subsequent treatment with larvicides is a well-established yet highly labour intensive process, However, LSM has diminished in prominence in malaria control programmes in the past two decades with the rapid scale-up of ITNs (insecticide-treated nets) [32, 33]. The emergence of relatively inexpensive commercial drones may reenergize efforts to target larval breeding sites by reducing the time and labour demands of identifying and treating these often remote locations. The high mobility of the aerial platform allows them to quickly traverse difficult terrain which would be difficult to access by foot or ground vehicles [34].

For mosquito breeding sites to be targeted effectively in disease control programmes, up-to-date information on their locations must be collected [29]. UAVs are multi-purpose platforms to which a variety of sensory equipment can be mounted. Based on user preference, UAVs can utilize passive sensors that capture reflected rays of electromagnetic radiation such as thermal, near infrared or visible spectrum, or combinations thereof in hyperspectral cameras [27, 34, 35]. Additionally, these systems may incorporate active sensors, such as radar or lidar which characterize the three-dimensional structure of terrain and vegetation. The specifics of these sensors have been extensively reviewed elsewhere [36]. For the purpose of this review, it is important to note that the visible spectrum cameras are usually part of commercially available UAVs and allow quick analysis of distinct water bodies that serve as mosquito breeding sites in the environment. Water that is muddy or covered by vegetation may be missed by visible-spectrum sensors [37], however hyperspectral sensors can detect water bodies by their thermal signature [36]. The detection of mosquito breeding sites with UAVs has also been investigated in urban environments, with the mobility of drones used to observe rooftops that may otherwise be inaccessible to technicians [38]. However, the acceptability of UAVs to local communities is important to assess prior to their deployment in the field, with cross-sectional studies of the general population undertaken in Malaysia, Mexico, and Turkey [28] to assess public views.

Recent studies have used UAVs to detect water bodies for larval source management and analysis of the best routes for teams to access them for malaria control in Zanzibar [27], Côte d’Ivoire [20], Malawi [34], Peru [39] and for detection of oviposition sites of arbovirus vectors in Mexico [38], Peru [40], Sri Lanka [41], and Australia [35]. Additionally, UAVs have been used in Borneo to investigate the movements of humans and primates to elucidate the transmission mechanisms of *Plasmodium knowlesi* malaria [19], as well as to map snail habitats to investigate the risk of schistosomiasis [42, 43]. However, despite the growing capabilities of UAV sensors for detecting vector habitats, there will remain a need for ground truthing to validate the classifications made by drone-captured images.

Prior to the introduction of UAVs for mapping mosquito breeding sites, satellite imagery and mapping using manned aircraft have been utilized for this purpose [44, 45]. Each of these tools offers advantages and disadvantages when compared to use of UAV, as summarized in Table 2. Aerial vehicles offer significant advantages in terms of the resolution of their imagery equipment [25] and their ability to operate below the cloud cover [34]. Satellite image timing can rarely be determined by the needs of the end-user and is often limited by cloud cover [46–48]. An analysis of satellite imagery (Landsat and Sentinel-2) available of a study area in Zanzibar showed that only two images out of 81 centred over the study area had lower than 5% cloud cover and could be considered useable and that none of the assessed images were completely cloud free [27]. However, UAVs have a number of limitations that satellites do not, such as their susceptibility to windspeed and precipitation, limited battery life and resulting flight range, as well as their dependence on manual control by a human operator. The dependence of UAVs on favourable weather conditions has implications for their utility in malaria control, as the peak of vector abundance is typically during the rainiest months of the year. Manned aircraft are somewhat less susceptible to weather conditions compared to UAVs, without the same restricted operational ceiling or need to maintain line-of-sight with a ground operator [27]. Additionally, the typically larger airframe and fuel capacity of manned aircraft allows for a longer operational time compared to smaller commercial UAVs used for vector control [49].

**Table 2:** Comparison between satellite, aerial and UAV systems for vector surveillance

Parameter	NASA/ESA satellites	VHR satellites	Aerial mapping	Fixed wing UAV	Rotary UAV
Revisit time	~ 5 days	~ 3 days	^a^	^a^	^a^
Resolution	~ 10 m	~ 0.3 m	~ 0.1 m	~ 0.01 m	0.015 m
Costs of imagery	Free	High	High	Low	Low
Area coverable	High	High	Medium	Medium–low	Low
Wind resistance	N/A	N/A	High–medium	Medium–low	Low
Influence of cloud cover	High	High	High	Medium–low (If no rain)	Low (if no rain)
Volume of data collected	Moderate	High	High	High–very high	High–very high

*NASA* North American Space Agency, *ESA *European Space Agency, *VHR  *very high resolution, *UAV  *unmanned aerial vehicle ^a^High, but depending on availability of aircraft & pilot/operator in the area of interest.

Adapted and expanded from Müllerová [25]

At present the scope of UAVs in vector control is limited by local and national regulations on their operation. Typically, countries will set limits on the operational ceiling (maximum altitude) of UAVs, though this may vary greatly between countries. For example, UAVS are not permitted to fly above 45 m in Malawi but can operate as high as 120 m in Cameroon (a summary of drone laws in different countries can be found at www.drone-laws.com). These operational ceilings have important implications for habitat surveillance as this limits the area that can be captured in a single image. Additionally, UAV operators are typically required to maintain line-of-sight with their aircraft at all times. [50, 51]. In June 2017, UNICEF and the Malawian aviation authority established a 5,000 square km corridor that allows for testing of UAV systems up to altitudes of 400 m, with a further three UAV corridors since opened in Vanuatu, Kazakhstan, Sierra Leone and Namibia [16, 52, 53]. These testing sites provide an opportunity for the effectiveness of UAVs to be assessed alongside conventional vector control techniques.

### Data processing

The restrictions on maximum altitude and requirements for high-resolution imagery outlined above result in a need for image stitching, which is demanding in terms of computer storage and power though the techniques for this process are subject to constant improvement [34, 54]. The images obtained by UAV-mounted sensors are typically stitched together into an assembled digital map (referred to as an ‘orthomosaic’) by using software such as Agisoft PhotoScan (www.agisoft.com) or Pix4D (www.pix4D.com). This digital map can then be uploaded into GIS (Geographic Information System) software, which can be geographically referenced using GPS (Global Positioning System) data automatically gathered by the UAV in flight if it possesses the appropriate sensors [34, 54]. Should a UAV lack an onboard GPS, geographic coordinates can be manually obtained by reference to Ground Control Points (physical ‘landmarks’ with known coordinates) [55].

**Fig. 2:**
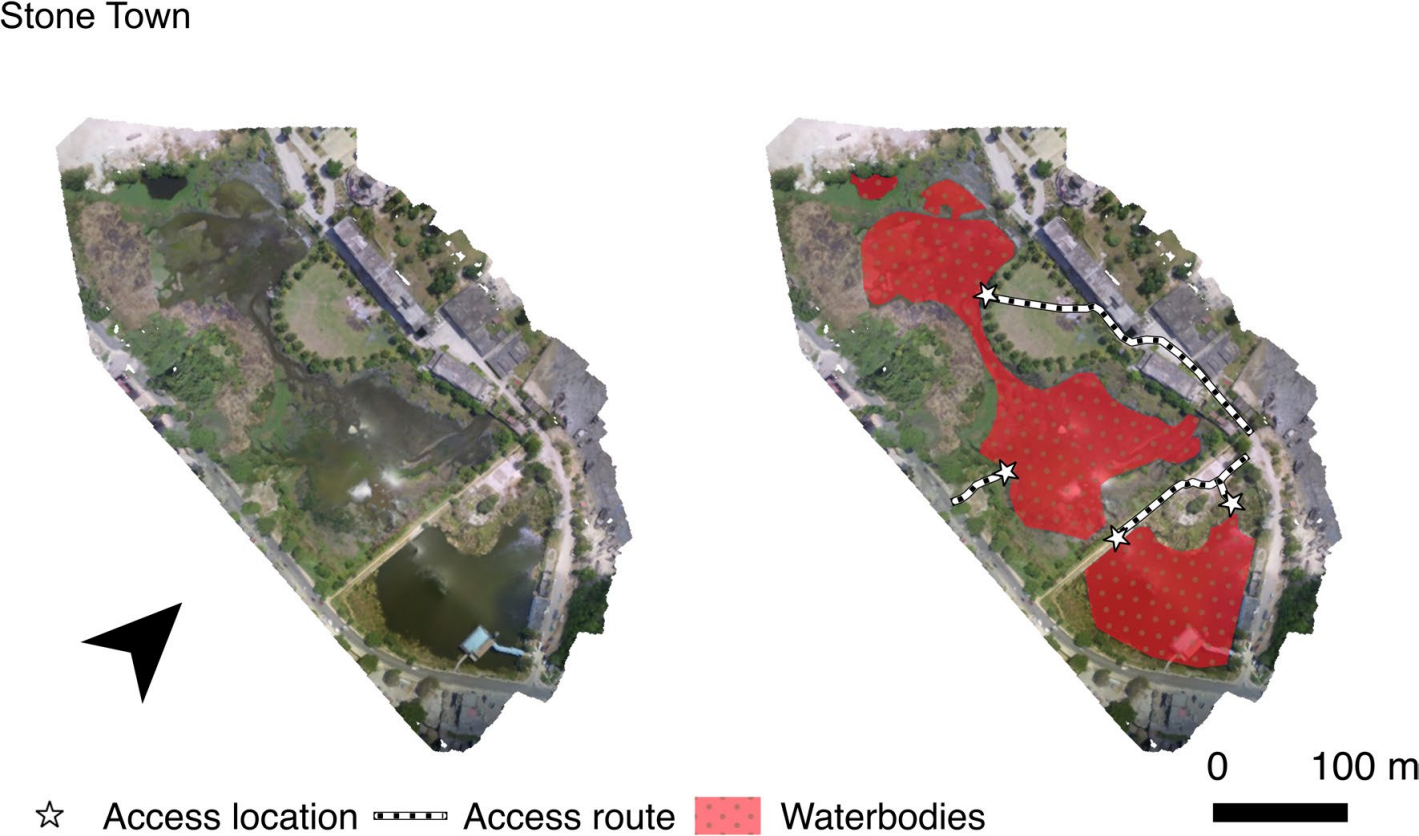
An example of **A** orthomosaic after stitching and **B** a map that can then be passed to field teams with highlighted waterbodies (red) and access routes and locations. Taken with permission from Hardy et al. [27]

The high-resolution digital maps obtained by UAV sensors can be used to inform a number of activities in vector surveillance and control. Image processing of the digital map may be performed manually by the user, or automatically by image processing software that classifies ‘objects’ (such as water bodies, aquatic vegetation, roads or human dwellings) [7, 56, 57]. The algorithms that perform these classifications are undergoing constant improvement [58] however their accuracy decreases should images include landscape features that they have not be trained on, which would instead require oversight by a human operator [34]. Once the processing is finished, the resulting map, called a prescription map, can then be uploaded to a UAV for the application of larvicides or other control measures; see Fig. 2 for an example of such a map. It should be noted that the digital files associated with UAV images may be very large (up to 70 terabytes), particularly with the high resolution required for accurate object classification [57]. Transferring and storing this data may pose practical challenges, as rural field sites may lack internet connectivity or even access to electricity [34, 59]. It is important to state that multi- and hyperspectral sensors require calibration and atmospheric corrections in order to avoid artefacts that impact the quality of data as well as to allow data comparison between flights [27]. Furthermore, the quality of images taken by a UAV may vary with the experience of the operator [34].

## UAVs for deploying vector control payloads

The use of UAVs which carry or deploy chemicals is widespread agriculture. A recent meta-analysis showed that most articles published on the use of UAVs were in agriculture, followed by geomorphology [60]. However, this technology is readily applicable to malaria control programmes. A number of current vector control interventions involve the delivery of material (such as insecticide or other control agent) to a target site. In principle, the use of UAVs as a delivery platform for these materials makes it more feasible for the currently labour-intensive techniques described below to be deployed precisely at scale [16, 61].

### Larvicide delivery

The growing role of UAVs in mapping mosquito breeding sites is accompanied by examples of their application to directly deploy larvicides to such water bodies [61]. The earliest example of UAVs transporting and deploying larvicide for control of mosquito vectors of infection was in Louisiana, United States in 2016 following cases of West Nile and Zika virus. A single Da-Jiang Innovations “Agras MG-1” octocopter designed for deploying herbicides in agriculture [62, 63] was used to deploy larvicide across hundreds of acres. Practical examples of the use of UAVs for malaria control in endemic countries are still in their early stages, however a recent study in Zanzibar, Tanzania demonstrated promising results, achieving a 90% reduction in *Anopheles arabiensis* density five weeks after treatment [61].

### Adulticide delivery

Insecticide space spraying, the dispersal of a liquid ‘mist’ to kill adult insects is a well-established technique in both agriculture and vector control programmes [64]. Spatial adulticides can be deployed via a number of delivery methods, such as handheld, vehicle or aircraft-mounted release equipment [65]. Space spraying is not typically considered as a routine control intervention for malaria and is instead used as an emergency tool in response to epidemics. However, it should be noted that a recent systematic review concluded that there is a lack of evidence to assess whether adulticide space spraying impacts malaria transmission [64].

The use of UAVs to transport and release adulticides for malaria control has not yet been assessed however a small number of investigations have been conducted for arbovirus control. The high mobility of the UAV allows it to access remote areas and directly target human structures or other terrain where mosquitoes are likely to converge. While aerial space spraying by manned aircraft is already an established method for treating large areas, UAVs provide a level of low-altitude precision that optimizes delivery and prevents waste. Spatial spraying with UAVs has been investigated in China against *Aedes* spp. vectors of dengue, where a 4% permethrin 1% tetramethylfluthrin mixture was observed to reduce the human-biting density of *Aedes albopictus* by 66.9% 24 h after exposure (though the reduction after 48 h was just 9.2%) [23]. Another study, releasing deltamethrin from a UAV onto caged *Culex quinquefasciatus*, resulted in 100% mortality over 12 hectares [66]. Most recently, a study from Williams et al. has introduced and tested a modular UAV system capable of delivering both larvicides (granulated or liquid), or Ultra-Low Volume (ULV) adulticide spraying [67]. The issue of payload is critical in many agricultural applications with high spray rates (10–20 L/ha) but less critical in vector control application, where the spray rates can be a low as 0.15 L/ha for *Aedes* control [68]. In such circumstances it is generally the flight time, limited by battery life, that is the limiting factor rather than the payload being insufficient.

### Insect release

In addition to their use in deploying insecticides, UAVs can be used to release live insects at a target site for the purposes of disrupting wild vector populations. Primarily this technique is used to release insects of the same species as the target to interfere with reproductive success, however there are examples in agriculture of natural predators being released to control a pest species [69]. The sterile insect technique (SIT), the release of sterilized males into wild vector populations, is a well-established technique for reducing human-biting density [32, 70, 71]. However, the process of transporting the insects to numerous remote target sites by ground-based transport is laborious and slow [71]. To date, release of sterile males has not seen widespread use for malaria control due to low levels of demonstrated efficacy on malaria outcomes seen to date, in part caused by the poor mating competitiveness with wild males [71]. However, emerging techniques to release transgenic malaria vectors to disrupt wild populations may see a revival in the need for technologies that can precisely transport and deploy mosquitoes to target sites [72].

While the release of sterile males by UAVs has not yet been investigated in the context of malaria control, successful trials have been demonstrated for arbovirus vectors in South America. The delivery of *Aedes* mosquitoes for SIT has been successfully trialled in Brazil, with a single modified DJI M600 UAV releasing 50,000 mosquitoes per flight to achieve a high level of induced sterility in the wild population [73]. The same study noted the spatially even distribution of releases achieved by UAVs compared to ground-based release sites, a particular advantage given the low dispersal distances of *Aedes* spp. mosquitoes [74, 75]. However, some initial challenges of transporting and deploying *Aedes aegypti* from UAVs have been identified by subsequent pilot studies in Southern Mexico, with approximately 50% fewer sterile males observed in post-release sampling compared to ground-based releases [76]. The authors suggested that compaction and physical injury within the drone compartment may have greatly reduced the survival of released males and highlight the design of release containers as a priority for future aerial-release programmes. Additionally, the same study indicated that the chilling of sterile males (4 °C for 20 min) to facilitate loading into drone release tubes may have reduced survival.

A similar project for *Glossina* (tsetse fly) control in Ethiopia focuses on the release of sterile tsetse flies in order to control human sleeping sickness. Unlike the octocopter used in the *Aedes* trial, a fixed wing UAV capable of dispersing 5000 tsetse flies over an area of 100 km^2^ (10,000 hectares) is being used [77]. The main difference between these organisms and other vectors including mosquitoes is that tsetse flies exist at very low densities and, therefore, require much fewer sterile male releases to achieve population suppression [78, 79]. Tsetse flies are larger and more robust insects with greater dispersal capacities compared to anophelines, thus a lower density of released insects is required to achieve suppression, and thus greater coverage is possible with the same rearing and release capacity [79]. Additionally, the habitat which tsetse *Glossina* flies occupy is usually rural and less densely populated, reducing the need for precision manoeuvring for which multirotor UAVs are more capable than fixed wing UAVs [80].

## Considerations in selecting the appropriate UAV

Commercially available UAVs present a variety of choices, each providing different capabilities and limitations for use in vector control (Table 3) [81]. Broadly, these can be divided into multi-rotor and fixed-wing designs [82, 83]. Multi-rotor UAV systems (such as the DJI Phantom) use horizontally orientated lift surfaces to allow them to hover. This hover capability makes these craft highly agile and allows them to take off from very small spaces as a result of VTOL (vertical take-off and landing) [80]. However, multi-rotor UAVs are typically limited to light payloads, no more than 2 kg. Fixed-wing UAVs (such as the SenseFly eBee) obtain lift from their large wing surfaces, providing them with greater flight endurance and payload capacity compared to multi-rotor designs. However, as their lift is provided by air moving over the wings, they require space to take off (roughly 100 × 20 m for the eBee) [62]. An interesting avenue in future UAV development is demonstrated by US company VAYU [32] that has created a hybrid UAV capable of vertical take-off and landing, but with wings that allow it to fly like a fixed wing aircraft [82, 84], reaching distances of up to 100 km. However, the aircraft is designed for the transport of biological samples, mechanical parts, or blood products and currently only offers a 2 kg payload capacity with no spray delivery system.

**Table 3:** Comparison of the use cases and available UAVs for vector control

Purpose	Design requirements	Opportunities	Limitations	Examples (cost USD)
Habitat surveillance	Sensor mounts	• Can be performed by inexpensive models	• Light designs unable to operate in wet/windy conditions	• DJI Phantom 4 ($900) • SenseFly eBee ($23,500)
Insecticide deployment	Payload capacity	• Technology readily adaptable from agriculture • High precision application • Can deploy insecticide to inaccessible terrain		• DJI AGRAS MG-1 ($13,000) • Yamaha FAZER R ($100,000)
Insect release	Release device	• Low payload requirement • Faster and more even distribution than ground release	• Compaction and chilling for drone release reduces survival	• DJI Matrice 600 ($6500)

## Regulations on the use of UAVs for malaria control

The development of civilian and commercial operation regulations for UAVs has been slow and, in certain cases, cumbersome [85, 86]. However, regulatory bodies have been catching up with the available technology in recent years, spreading the regulatory responsibility among different authorities. The rules and regulations governing UAV operations are ever-changing and can vary greatly between countries. Regulations may be clearly established in some places, as in the EU [87] and USA [88], yet where regulations have not yet been established disputes and even confiscations of equipment may arise [85]. Operational parameters commonly limited by aviation authorities are: distance from the operator, payload and release, altitude, and proximity to buildings and centres of the human population [86]. The Drone Regulations Project provides publicly-available information on regulations for various countries [89], though local use exemptions may apply and it is advisable still to seek official documents and permissions prior to commencing any operations. Regulations may also add hidden costs to UAV operations as certain countries may require license fees or costly local operator courses [34]. Perceptions of UAV use by the public may also vary from place to place [90, 91], though key concerns tended to focus on physical safety and privacy. Consequently, it is advisable to conduct community engagement work prior to commencing operations as well as to include information on drones in broader educational materials on disease control [28]. However, ethical concerns regarding the impact of UAVs and associated data on safety and privacy cannot be wholly addressed by community engagement and educational materials, with a need for clear laws and regulations that limit the opportunities for adverse impacts to occur. A recent review by Lee et al. stresses the importance of opt-out rights for residences, disclosure of data management practices, specified limitations on third party sharing of data, as well as trusted agencies to monitor adherence to regulations and enforce penalties for violations [92].

## Discussion

UAVs are emerging as a potentially useful addition to the current toolkit for malaria vector control, especially in elimination and eradication settings where transmission is more likely to be concentrated in specific hotspots that require frequent surveillance and treatment [93]. Furthermore, UAVs are proving promising in accessing remote or difficult terrain, such as dense forestry, marshlands or urban landscapes. The ability of some UAVs to carry and deploy payloads makes them suitable for the release of control agents at target locations that might otherwise be inaccessible to conventional ground-based transport [13, 22, 27, 76]. There are widespread examples of UAV use in vector habitat surveillance, with the low cost and small take-off space required offering advantages compared to much larger manned aircraft. Additionally, the capability to operate below cloud cover addresses a key disadvantage of remote sensing by satellite data, with the caveat that UAVs typically cannot be flown in high winds and rain. Given the rapid technological development and expanding use of UAVs in agriculture for monitoring pest species and plant health as well as a wide array of pesticides, it can be expected that the applications for malaria control will continue to widen in the coming years. In particular, the growing market for UAVs that can transport and deploy large payloads of pesticides across extended flights for agricultural purposes may address the aforementioned issues of short range and payload capacity of UAVs currently used for insecticide spraying in malaria control. Furthermore, the continuing development of image processing algorithms to interpret images collected by drones can be expected to accelerate automation and reduce human labour requirements [34]. Given these growing data processing requirements, there is a need for individuals within national malaria control programmes (NMCPs) that possess the relevant data processing skillsets. In response to this growing demand, the African Drone and Data Academy was launched in 2020 in Malawi to provide high-quality courses in both drone operation and data visualization [94]. Additionally, networks of researchers such as MACONDO have been established to support operational research using UAVs and develop guidelines for their use[95].

A key challenge to the scaling up of UAVs for malaria control and wider global health applications is the perceptions and acceptance of at-risk communities. Understandably, communities may have concerns regarding their safety and privacy, highlighting a need for both a dialogue with malaria control programmes and clear regulations on their use. While few examples of community engagement in the context of UAVs for malaria control exist currently, a recent study in Zanzibar observed low rates of exposure or awareness of UAVs highlighting the need for clear, non-technical language to assess acceptability and obtain informed consent [96].

As UAV technology for global health is still an emerging area of research, the relative utility of this technology compared to established methodologies remains poorly understood. Cost comparisons between UAVs and standard methods for surveillance and control must be undertaken, and standardized methods of conducting such studies established [85]. At present, such comparisons are limited to contexts other than global health. A recent study comparing manned aircraft, satellite data and UAVs in surveillance for viticulture indicated that an economic break-even between UAVs and other platforms exists in between 5 and 50 ha of coverage [97]. However, this comparison was conducted in 2014 and in a different setting to vector control thus studies on cost effectiveness of UAVs in vector control is urgently needed to accommodate different environments and assess developments in the technology.

To assess the viability of scaling of UAV use for vector surveillance and control, there is a need to establish their relative fuel efficiency compared to conventional ground transport based techniques, particularly as fuel and electricity availability becomes a growing international concern. While such data is not available for the specific context of global health, a recent *in silico* simulation study on the efficiency of UAVs for domestic delivery found multirotor UAVs were less energy intensive than diesel trucks when the number of stops in each flight was low however UAVs tended to become increasing less efficient as the number of stops increased and as well as being with increasing windspeed [98]. Additionally, access to electricity and internet pose practical challenges to operating in the field.

In conclusion, UAVs are emerging as a potential useful addition to the malaria control toolbox, with their high mobility facilitating surveillance of vector habitats and delivery of vector control payloads across difficult terrain. However, there is a need for both further research on the cost-benefit of UAVs compared to existing techniques and the development of frameworks to both permit and regulate their use in endemic settings. The willingness to pay for the inclusion of UAV-enhanced vector surveillance or control will depend on this cost-benefit analysis, and will likely be situation dependent based on the specific activity and the funders.

## Data Availability

Not applicable.

## Abbreviations

AMCA: American Mosquito Control Association
BVLOS: Beyond visual line of sight
CAA: Civil Aviation Authority
EASA: European Union Aviation Safety Agency
ESA: European Space Agency
FAA: Federal Aviation Administration
GCP: Ground control point
GPS: Global positioning system
IRS: Indoor residual spray
IVCC: Innovative Vector Control Consortium
LLIN: Long lasting insecticide net
NASA: North American Space Agency
NIR: Near infra-red
RC: Remote control
RPAS: Remotely piloted aircraft system
RTK: Real-time kinematic GPS
SIT: Sterile insect technique
UA: Unmanned aircraft
UAS: Unmanned aerial system
UAV: Unmanned aerial vehicle
VBD: Vector-borne diseases
VHR: very high resolution
VTOL: Vertical take off and landing
